# Dynamic monitoring of single cell lysis in an impedance-based microfluidic device

**DOI:** 10.1007/s10544-016-0081-z

**Published:** 2016-06-14

**Authors:** Ying Zhou, Srinjan Basu, Ernest D. Laue, Ashwin A. Seshia

**Affiliations:** 1 Department of Engineering, Nanoscience Centre, University of Cambridge, 11 JJ Thomson Avenue, Cambridge, CB3 0FF UK; 2 Department of Biochemistry, University of Cambridge, 80 Tennis Court Road, Cambridge, CB2 1GA UK

**Keywords:** Microfluidics, Single cell trapping, Impedance spectroscopy, Stem cells, Real-time measurements

## Abstract

A microfluidic device that is capable of trapping and sensing dynamic variations in the electrical properties of individual cells is demonstrated. The device is applied to the real-time recording of impedance measurements of mouse embryonic stem cells (mESCs) during the process of membrane lysis, with the resulting changes in the electrical properties of cells during this process being quantitatively tracked over time. It is observed that the impedance magnitude decreases dramatically after cell membrane lysis. A significant shift in the phase spectrum is also observed during the time course of this process. By fitting experimental data to physical models, the electrical parameters of cells can be extracted and parameter variations quantified during the process. In the cell lysis experiments, the equivalent conductivity of the cell membrane is found to increase significantly due to pore formation in the membrane during lysis. An increase in the specific capacitance of the membrane is also observed. On the other hand, the conductivity of the cytoplasm is observed to decrease, which may be explained the fact that excess water enters the cell through the gradual permeabilization of the membrane during lysis. Cells can be trapped in the device for periods up to several days, and their electrical response can be monitored by real-time impedance measurements in a label-free and non-invasive manner. Furthermore, due to the highly efficient single cell trapping capacity of the device, a number of cells can be trapped and held in separate wells for concurrent parallel experiments, allowing for the possibility of stepped parametric experiments and studying cell heterogeneity by combining measurements across the array.

## Introduction

It has been recently expounded that individual cells, even those identical in appearance and type, would have different characteristics and have an unpredictable and heterogeneous behaviour in a given population (Di Carlo 2009). The cell-to-cell variability, also termed as cellular heterogeneity, can result from the intrinsic stochasticity of gene expression or from extrinsic factors such as the heterogeneity of the surrounding microenvironments (Huang 2009). Due to these cell-to-cell variations, traditional biochemical assays, which analyse cells in bulk and yield data averaged across large cell populations, often overlook information about single cell behaviour and the cell heterogeneity within a population, and this could lead to misleading interpretation of experimental results for such systems (Di Carlo 2009; Sims and Allbritton 2007; Svahn and Berg 2007).

To identify the cell-to-cell variability in a population, the analysis of individual cells is mandatory. Single cell analysis can effectively remove most uncertainties caused by bulk techniques such the loss of information inherent in data averaging (Svahn and Berg 2007). Over the past few decades, efforts have been made to develop techniques that enable the study of individual cells as well as the heterogeneity of cell populations. A conventional technique for analysing cell populations at single-cell resolution is flow cytometry. However, conventional flow cytometry only provides the instant information about cell distributions within a population, but does not provide the real-time or time-dependent information about how individual cells behave and change with time.

The interest in obtaining time-dependent information on the behaviours and dynamic processes of individual cells (such as cell cycle, differentiation and lysis) has seen much recent interest. Microfluidic techniques are often best suited to this purpose (Sun and Morgan 2010) with the potential for samples of interest to be confined in micro−/nano-metric spaces for detection, and enabling the manipulation of target analytes within microscale channels. Such miniaturised devices provide unique platforms for studying and characterising the activity of individual cells and molecules. One common approach to realise single cell trapping is to add micromachined structures (e.g. micro-posts, micro-pillars, or micro-traps) in the microchannel to capture cells based on hydrodynamic flow (Chen et al. 2013; Di Carlo et al. 2006a; Di Carlo and Lee 2006; Di Carlo et al. 2006b; Frimat et al. 2011; Jin et al. 2015; Kobel et al. 2010; Kumano et al. 2012; Nilsson et al. 2009; Skelley et al. 2009). This approach, also referred to as mechanical trapping or hydrodynamic trapping, employs MEMS technology to fabricate micro-mechanical traps in the channel for trapping samples. In particular, previous work by Tan et al. outlined a highly efficient hydrodynamic approach to immobilise large numbers of beads in stable trap configurations, with each trap sequentially connected using a looped microchannel (Tan and Takeuchi 2007; Tan and Takeuchi 2008).

Electrical impedance spectroscopy is a non-invasive and label-free technique for cell characterisation, based on the measurements of dielectric/impedance properties of cells (Sun and Morgan 2010). These dielectric/impedance properties, considered as electrical markers of cells (i.e., physical markers from electrical measurements), can be used to quantify the physical state of cells. Such label-free techniques not only allow the identification of the cells for which the specific biological markers are known, but also allow the identification of those whose specific markers are currently not fully understood. Microfluidic platforms for single-cell impedance flow cytometry have previously been reported (Chen et al. 2014; Gawad et al. 2004; Gawad et al. 2001; Holmes et al. 2009; Morgan et al. 2007; Myers et al. 2013; Song et al. 2013; Zhu et al. 2014), where cells are driven into the sensing region of a microchannel in a continuous flow and their impedance is measured by microelectrodes embedded in the channel. These systems are typically operated in continuous-flow mode. However, it is difficult to measure the time-dependent response of an individual cell in a flow-through system. As mentioned above, there is often a requirement to monitor single cells over a significant time course so that their dynamic response may be studied. Therefore, integrating label-free impedance spectroscopy techniques with long-term single cell trapping schemes addresses this requirement. The main advantage of the single cell trapping/sensing technique is that it allows the change in impedance to be observed over time, which may be more easily correlated to a specific morphological development of the cells. Another advantage is that it enables a highly detailed impedance spectrum to be measured, which makes the procedure of extracting physical values for the dielectric parameters more reliable. Malleo et al. previously reported a glass-based microfluidic device containing U-shaped microstructures for cell capture and microelectrodes for impedance measurements, and studied the impedance change of Hela cells in response to chemical disruptions (Malleo et al. 2010).

In recent work (Zhou et al. 2016), we have presented a PDMS/glass-based microfluidic device, integrating hydrodynamic trapping and impedance sensing functions to study single cells. We have demonstrated that the device provides highly sensitive impedance measurements, and showed that cells at different differentiation states can be distinguished using the impedance-based approach. In this work, we apply this technique to perform real-time measurements of the electrical properties of mouse embryonic stem cells (mESCs) during induced cell lysis, verifying that this technique enables real-time single cell studies over a period of up to several days. Compared with Malleo’s work (Malleo et al. 2010) in which glass-SU8-glass based microchannels were used, we have employed a different trapping design and sensing configuration, which simplifies the fabrication process (e.g. using soft lithography techniques) and enables more efficient and reliable single cell immobilisation for impedance measurements. The impedance spectra of individual cells over a wide frequency span is recorded over the time course and changes in the electrical properties of cells are dynamically tracked during the cell lysis process. Measuring the entire frequency response of cells and the subsequent analysis of the response enables the electrical parameters of cells to be extracted. A quantitative analysis is performed to understand how the electrical properties of cells change during lysis. The technique and methodology described in work could be beneficially applied to further research on single cells, where characterization and selective separation of targets cells from cell mixtures are required.

## Methods

### Design and simulation

**Fig. 1:**
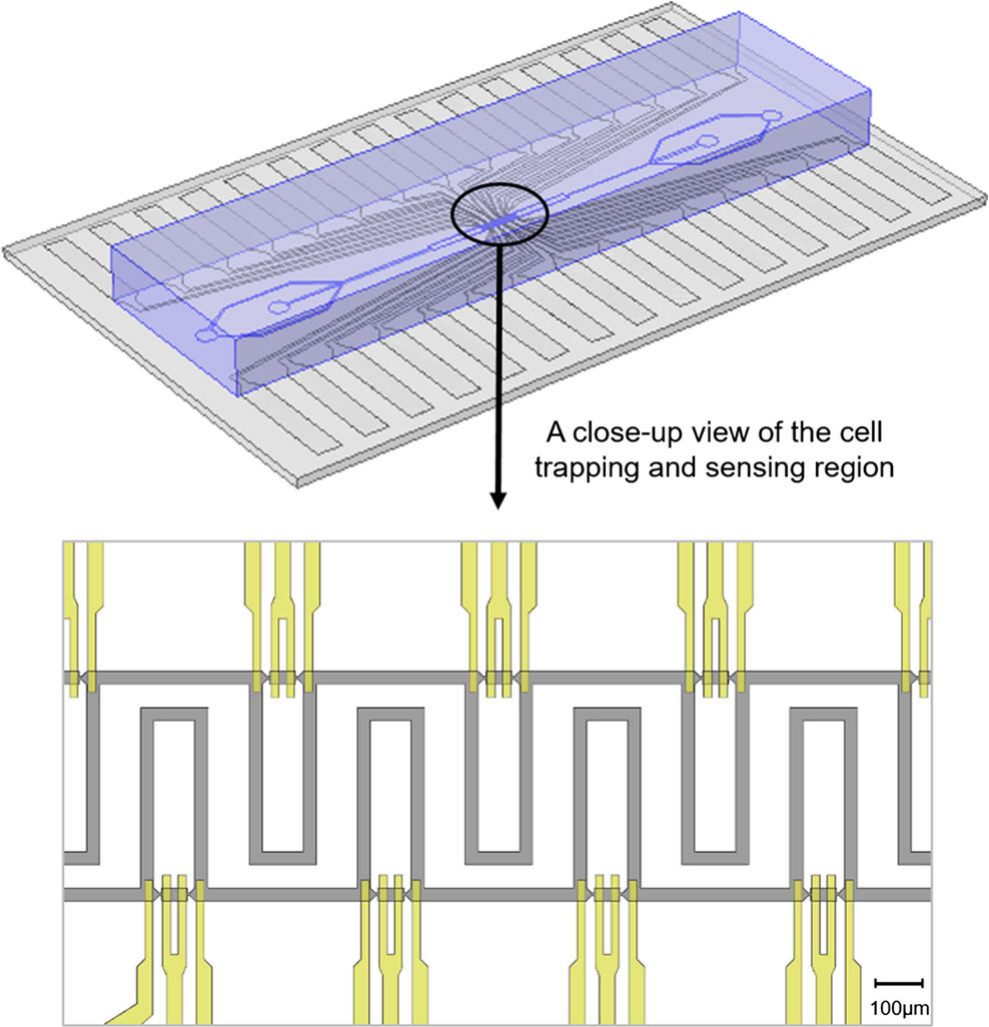
A 3D schematic diagram of the microfluidic device with integrated coplanar electrodes is shown above. A close-up view of the cell trapping and sensing region in the device is provided below, illustrating the design of microfluidic trapping channels (grey colour) and impedance sensing electrodes (yellow colour). Scale bar is 100 μm

Figure 1 shows a 3D schematic diagram of the device, as well as a close-up view of the cell trapping and sensing region. The device is composed of a PDMS (polydimethylsiloxane) layer containing microfluidic trapping channels, and a glass substrate patterned with sensing electrodes. The two pieces are aligned and bonded together to form the final device, which is capable of immobilising single cells and performing electrical impedance measurements. The design of microfluidic trapping channels (grey colour) and impedance sensing electrodes (yellow colour) is illustrated in the zoom-in fig. at the bottom.

**Fig. 2:**
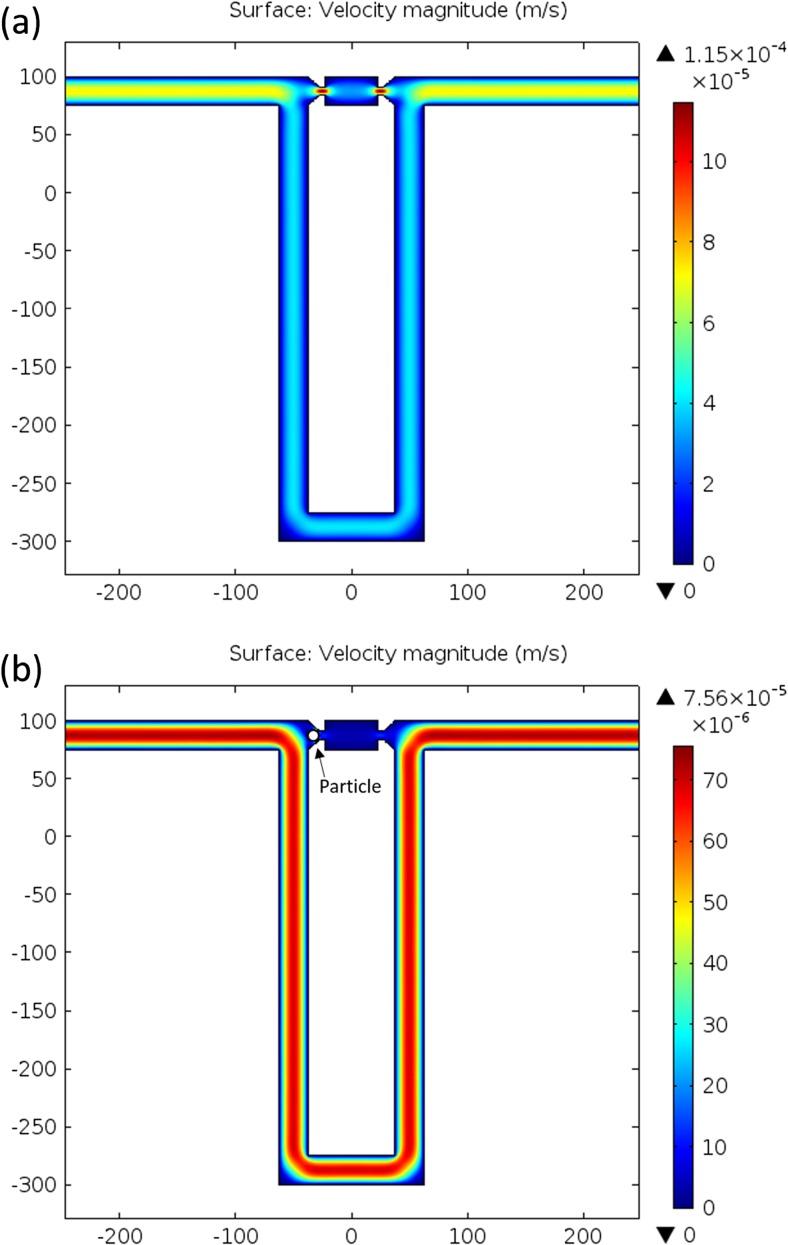
COMSOL simulations showing the flow velocity in the trapping channels: (a) when a trap is empty; (b) when a trap is occupied by a 10 μm particle

Single cell trapping is realised based on hydrodynamic flow (Tan and Takeuchi 2007; Tan and Takeuchi 2008). The detailed design of the trapping channels is shown in Fig. 2. There are two flow paths in each trapping unit: a short and straight path containing a narrow trapping gap smaller than the size of a cell to mechanically immobilise the cell; and a long and looped bypass channel, which could shunt cells to other trapping sites if the current trap has already been occupied by one cell. Fluidic resistance (or hydrodynamic resistance), a commonly used term in the analysis of fluid hydrodynamics, is defined as *R*_*c*_ = ∆*p*/*Q*, where ∆*p* is the pressure drop along a channel and *Q* is the volumetric flow rate in the channel. Flow resistance is a measure of the resistance to fluid flow (an analogy to the electrical resistance which is a measure of the resistance to current flow in the electrical energy domain), and is related to the geometry of channels and properties of solution. Due to the length and geometry difference between the two paths (as shown in Fig. 2), the flow resistance along the short channel can be designed to be smaller than that along the bypass channel. This results in a greater flow rate in the short channel, which can efficiently drive a cell into an empty trap. Once a trap is occupied by a cell, the flow path in the short channel is blocked by the trapped cell and other cells are thus directed to the bypass channel and driven to the next available traps by hydrodynamic forces.

For efficient single cell trapping, the volumetric flow rate along the bypass channel should be smaller than that of short trapping path (Tan and Takeuchi 2007). The volumetric flow rate in both fluid channels can be derived from the Darcy–Weisbach equation and momentum equations for the Hagen–Poiseuille flow, which quantitatively describes the relationship between the flow velocity or pressure drop along channels and the geometric dimensions of the fluidic channels. Depending on the above-mentioned criterion, the geometric dimensions of the channels are designed for capturing cells whose size is in the range of 5 μm to 20 μm in diameter. The geometric dimensions are summarised below: the width and length of the trapping gap are both 5 μm; the width and length of the bypass channel are 25 μm and 805 μm, respectively; the width and length of the middle chamber between the two oppositely facing traps are 25 μm and 50 μm, respectively; the height of the channels is 25 μm.

Finite element simulations have been carried out to study the flow velocity profiles in the channels (Fig. 2). The creeping flow module in COMSOL 4.4 is used. The fluid-flow simulation solver is based on the Navier–Stokes equations. The flow is assumed to be compressible (Ma < 0.3); inlet velocity is set to be 100 μm/s; outlet pressure is set to be 0 Pa; walls are set to the ‘No slip’ wall boundary condition.

When a trap is empty, the flow velocity in the small trapping gap is much higher than the surrounding liquid, as illustrated in Fig. 2a, thereby driving particles into the trap. A particle tracing simulation has also been carried out to study particle trajectories in the channels and the probability of particles flowing into the trap. One hundred particles (diameter = 10 μm; density = 1050 kg/m^3^) are released at the inlet of the channel and driven by the drag force of the fluid. According to the particle tracing simulation, the transmission probability of particles flowing into the trap is 52 %, which is greater than the probability for the particle to flow into the bypass channel, i.e., 48 %. These transmission probabilities indicate whether the trapping is efficient or not. The criteria for efficient single particle trapping is that the probability of particle flowing into the trap should be larger than that of particle flowing into the bypass (Tan and Takeuchi 2007). The simulation results show that the proposed design satisfies the criteria for efficient single particle trapping.

The simulation result in Fig. 2b shows how the flow profile is modified when a particle is being trapped. Once a trap is occupied by one particle (10 μm diameter in simulation), the flow path through the short straight channel is blocked by the trapped particle. There is no flow through the trapping gap (i.e., flow velocity is nearly zero) and the only flow path remaining is through the bypass channel. A time-dependent particle tracing simulation has been performed, verifying that no particle is driven into the trap when it is already occupied by one. According to the simulation, the probability of particles flowing into the trap is almost zero, while the probability for the particle to flow into the bypass channel is 100 %. This implies that when a trap is filled with a particle, other particles will be driven to the bypass channel by the drag force of the fluid and then to the next available trap.

**Fig. 3:**
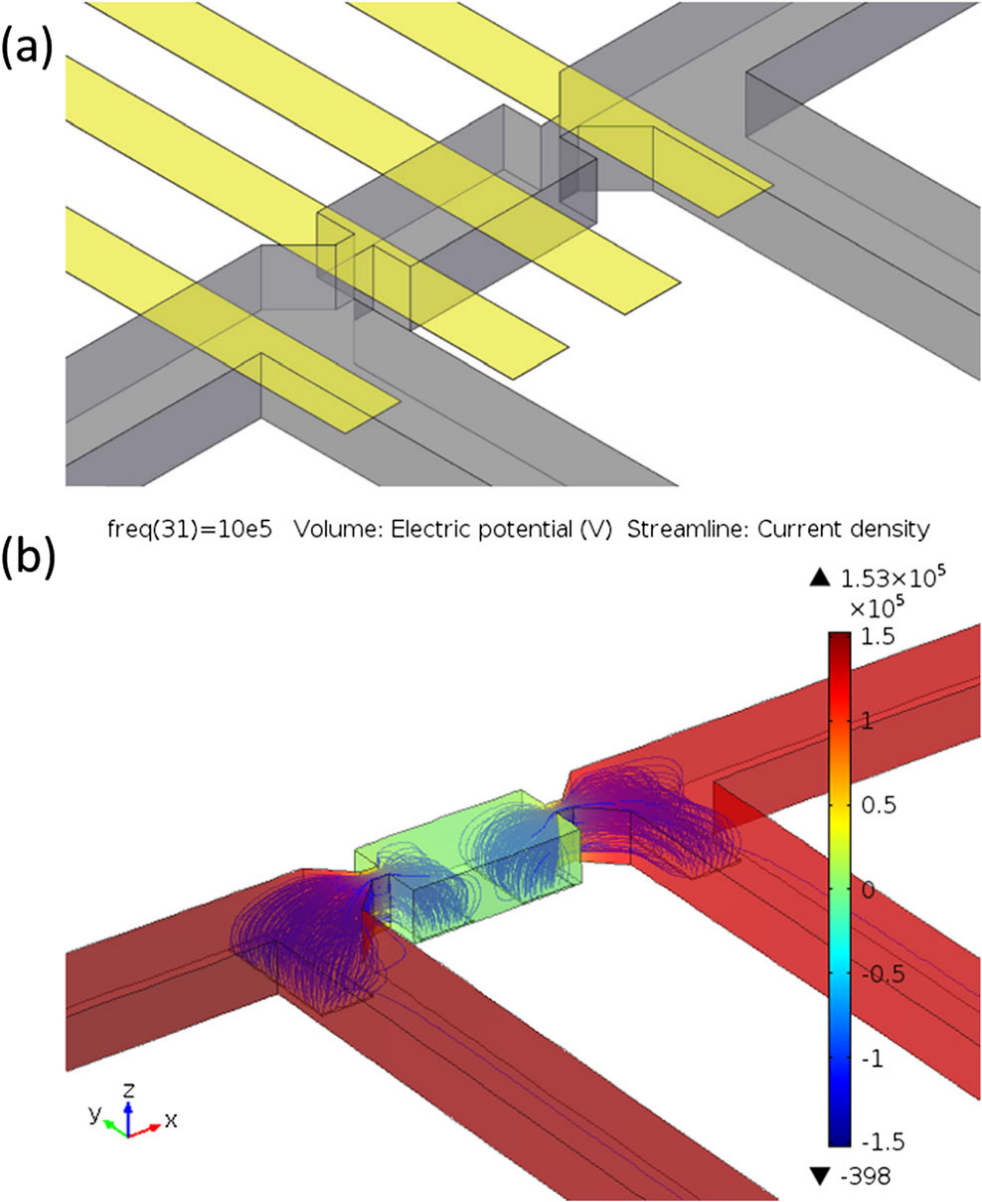
Finite element model and simulation of the electric field inside the trapping device. (a) 3D schematic view of a trapping and sensing unit. Microfluidic trapping channels (grey colour) and impedance sensing electrodes (yellow colour) are shown. (b) Simulation showing the electric potential (with colour bar) and current density (streamlines) inside the channels

The schematic diagram illustrating the detailed electrode configuration is shown in Fig. 3a. In one trapping group, there are two traps sitting closely but facing oppositely to each other. Each trap site consists of two sensing electrodes. The width of the electrodes is 15 μm, and the distance between two neighbouring electrodes is 15 μm. Depending on the flow direction, cells will be captured at the trap facing upstream, whereas the trap facing downstream is empty. The two electrodes below the trap where a cell is trapped are known as the sensing electrodes, while the other two electrodes measuring the impedance of the empty trap are known as the reference electrodes. A differential spectrum can be obtained by comparing the impedance measured from the sensing electrodes with that measured from the reference electrodes to cancel out any common-mode effect caused by unexpected drifts in measurement conditions or electrode properties (Gawad et al. 2001). Figure 3b shows the simulation results from finite element analysis (COMSOL 4.4 AC/DC module), demonstrating the electric field and current density distributions in the channels. Because of the narrow trapping gaps that are structured in the channel, the current density in the narrow trapping gap is found to be highest, as demonstrated by the region with the most concentrated streamlines. A cell, immobilised by the trapping gap, will modify the electric field and current streamlines between the sensing electrodes and thus lead to an impedance change. Quantitative analysis of the impedance change based on physical models will be discussed in the next section.

### Double-shell cell model

The double-shell cell model, where “double-shell” implies the cell membrane and nuclear envelope, was first described by Irimajiri et al. (Irimajiri et al. 1978; Irimajiri et al. 1979). In this model, a cell is composed of a cell membrane, cytoplasm, nuclear envelope and nucleoplasm. Assuming a cell is suspended in a medium, the equivalent complex permittivity of the whole mixture (cell and medium) is given by the Maxwell’s theory of interfacial polarisation:

$$ {\tilde{\varepsilon}}_{mix}={\tilde{\varepsilon}}_{med}\frac{2\left(1-\varphi \right)+\left(1+2\varphi \right){\tilde{\varepsilon}}_{cell}/{\tilde{\varepsilon}}_{med}}{\left(2+\varphi \right)+\left(1-\varphi \right){\tilde{\varepsilon}}_{cell}/{\tilde{\varepsilon}}_{med}} $$

where ${\overset{\sim }{\varepsilon}}_{mix}$ is the equivalent complex permittivity of the cell-medium mixture, ${\overset{\sim }{\varepsilon}}_{cell}$ is the equivalent complex permittivity of the cell, ${\overset{\sim }{\varepsilon}}_{med}$ is the complex permittivity of the suspending medium, and *φ* is the fractional volume of the cell relative to the suspending system. The complex dielectric permittivity is described as: $\overset{\sim }{\varepsilon}\left(\omega \right)=\varepsilon -j\sigma /\omega$, where *ε* is the absolute permittivity, σ is the conductivity and *ω* is the angular frequency. The expression of the equivalent complex permittivity of the cell (${\overset{\sim }{\varepsilon}}_{cell}$), containing a cell membrane, cytoplasm, nuclear envelope and nucleoplasm, has been derived based on the double-shell model and discussed in detail by Irimajiri et al. (Irimajiri et al. 1978; Irimajiri et al. 1979) and Asami et al. (Asami et al. 1989).

In the proposed microfluidic system, the complex impedance measured from the electrodes in the sensing group (containing cell-medium mixture) can be expressed as: ${\overset{\sim }{Z}}_{sense}=1/\left(j\omega {\overset{\sim }{\varepsilon}}_{mix}{G}_f\right)+2/\left(j\omega {C}_{DL}\right)$, where *G*_*f*_ is the geometric constant of the system (Sun et al. 2010; Sun et al. 2007) and ${\overset{\sim }{C}}_{DL}$ is the capacitance of the electrical double layer formed at the interface between the electrode surface and the electrolyte. On the other hand, the total complex impedance measured from the electrodes in the reference group (containing medium only) is given by: ${\overset{\sim }{Z}}_{ref}=1/\left(j\omega {\overset{\sim }{\varepsilon}}_{med}{G}_f\right)+2/\left(j\omega {C}_{DL}\right)$. The differential spectrum of a cell can be obtained by normalising the impedance of the sensing group with respect to the impedance of the reference group (Malleo et al. 2010), i.e., ${\overset{\sim }{Z}}_{diff}={\overset{\sim }{Z}}_{sense}/{\overset{\sim }{Z}}_{ref}$. In order to eliminate the influence of device geometry mismatch and fabrication errors, the device is calibrated prior to cell trapping to obtain a baseline spectrum, ${\overset{\sim }{Z}}_{base}$, which corresponds to the value of ${\overset{\sim }{Z}}_{diff}$ when no cell is trapped. A normalised spectrum, which is defined as the ratio of the differential spectrum of a cell to the corresponding baseline spectrum of the device (${\overset{\sim }{Z}}_{norm}={\overset{\sim }{Z}}_{diff}/{\overset{\sim }{Z}}_{base}$), can then be derived. The magnitude of the normalised spectrum is $\left|{\overset{\sim }{Z}}_{norm}\right|$, and the phase is *Φ*_*norm*_.

### Device fabrication

PDMS (polydimethylsiloxane) rapid prototyping technique was employed for fabricating the microfluidic channels. PDMS was chosen due to its low cost and other advantages such as its good elasticity and conformity. Firstly, SU-8 2025 (MicroChem) was patterned on a 3″ silicon wafer by standard photolithography, serving as the master mould for microfluidic channels. Before being used for PDMS moulding, the master mould was treated with FDTS (1H,1H,2H,2H-Perfluorodecyltrichlorosilane, 96 %, Alfa Aesar) by vapour deposition (Bell et al. 2014). The wafer was placed in a vacuum desiccator next to an open vial containing 100 μl of FDTS and 2 ml of hexane (as a solvent). The desiccator was pumped down until hexane was evaporated. After this, the desiccator was sealed and the wafer was incubated with FDTS vapour under vacuum overnight. The FDTS-coated surface is highly hydrophobic, which eases the PDMS peeling process from the master mould.

PDMS (Sylgard 184, Dow Corning) was spun onto the master mould at 500 rpm for 8 s and 1000 rpm for 60 s, and then cured on a 65 °C hot plate for 2 min and on a 150 °C hot plate for 5 min. The reason for using the PDMS spin coating method rather than the conventional pouring method is to address the shrinkage-induced PDMS alignment problem. Pouring PDMS directly onto a master mould typically results in a thick PDMS layer (> 1 mm in thickness). However, it has been observed that moulded structures in a thick-layer PDMS will shrink when the PDMS sample is peeled away from its master mould, due to thermal expansion and contraction during the curing process (Lee and Lee 2008; Moraes et al. 2009). The shrinkage of thick-layer PDMS causes problems when aligning PDMS with other substrates, and even makes it impossible to precisely align fine PDMS structures with other fine patterns. Furthermore, the degree of PDMS shrinkage and deformation may vary from batch to batch depending on many factors such as curing temperature and sample thickness, making it difficult to achieve repeatable results every time. Therefore, in this work, PDMS is spin coated onto the master mould. The resulting PDMS layer (about 60 μm in thickness) was thin enough that the shrinkage of PDMS can be neglected. It is found that this approach can effectively solve the shrinkage-induced PDMS registration problem associated with aligning thick-layer PDMS samples, and fine PDMS structures can be accurately aligned with electrodes patterns without shrinking or deformation using this approach. For easily handling the thin PDMS layer, a separate thick PDMS substrate was prepared by pouring the PDMS onto a blank wafer. After curing, the thick PDMS substrate was peeled off from the blank wafer, activated with oxygen plasma and bonded to the thin PDMS layer. The bonded sample was then peeled off from the master mould, after which inlets/outlets (1 mm diameter) were drilled by a biopsy punch.

Electrodes were fabricated by lift-off. AZ 5214E photoresist (MicroChemicals), used in image reversal mode, was patterned on a 2″ Pyrex glass wafer by photolithography, serving as a sacrificial layer for lift-off process. Metal electrodes (20 nm Ti and 100 nm Au) were deposited on the wafer by electron beam evaporation. Lift-off was performed in AZ 100 remover (MicroChemicals) bath at 50 °C.

The alignment and bonding of the PDMS channels and electrode patterns was done using a mask aligner. Prior to bonding, the bonding surface of PDMS was activated by oxygen plasma (Diener etcher, 100 % power for 20 s at 1.0 mbar). The back of the PDMS sample was attached to a 1 mm-thick custom-made acrylic adapter, whose surface is coated with FDTS to avoid PDMS adhesion, and the front surface of the PDMS sample, containing trapping channel structures, is exposed and available for bonding with the glass substrate with electrode patterns. The acrylic adapter, together with the PDMS sample, were loaded into the mask aligner and held by the vacuum mask holder of the mask aligner. The glass substrate was loaded into the wafer chuck of the mask aligner. The alignment process is similar to the standard process of aligning photo-masks and wafers during photolithography. Once the alignment of the microchannels and electrode patterns was done, the PDMS sample and glass substrate were brought into conformal contact to realise permanent bonding. The alignment and bonding process was typically completed within 15 min after the oxygen plasma treatment. The alignment errors using this method were typically smaller than 5 μm. After alignment and bonding, the device was post baked in a 65 °C oven for 1 h to improve the bonding strength. The electrical connections between the on-chip electrodes and external measuring instrumentation (Solartron SI 1260 impedance analyser) were made by using surface mount connectors that are bonded to the electrode pads on the chip using silver conductive epoxy.

### Sample preparation and experimental setup

Mouse embryonic stem cells were cultured in standard serum and LIF (Leukemia Inhibitory Factor) conditions (Reynolds et al. 2012). Cells were fixed in 2 % methanol-free formaldehyde for 5 min and then resuspended in PBS. The microfluidic device was connected to a syringe pump and the fluid flow was injected into the device via PTFE tubing. Before experiments, the microchannels were washed with 1 % BSA (bovine serum albumin) in PBS (phosphate-buffered saline) to prevent hydrophobic interactions between cells and PDMS channels. Calibration of the device was performed prior to cell trapping, serving as a baseline measurement. After the calibration, cells were loaded into the device at flow rate of 20 μl/h. After cells were trapped, the device was washed with buffer. Cell impedance measurements were carried out using an impedance analyser. The fluid flow was stopped during the impedance measurements to minimise cell deformation caused by the flow (cells were observed to stay at the trapping sites stably when the flow was stopped; they would only move out of the trapping sites when the flow direction was reversed). The input signal to the device is 100 mV, and the measurement frequency ranges from 100 Hz to 20 MHz.

## Results and discussion

An impedance-based experiment was carried out to quantitatively study the cell membrane lysis process and the change of cell electrical properties over the course of time. This study demonstrates the capability of the device in trapping and monitoring single cells for a given time course.

Malleo et al. (Malleo et al. 2010) studied the lysis of HeLa cells by measuring the cell impedance at 300 kHz and found a decrease in impedance magnitude at that frequency after the cell membrane was lysed by Tween 20 or SLO (Streptolysin-O). We carried out similar but extended analysis to study the lysis of mouse embryonic stem cells (mESCs), of which the size ranges from 8 μm to 12 μm. Instead of measuring cell impedance at a particular frequency, we monitored the real-time impedance spectra of individual cells over a wide frequency span (1 kHz ~ 10 MHz). The study of frequency response enables us to extract electrical parameters from the cells and perform quantitative analysis of the variation in electrical properties.

**Fig. 4:**
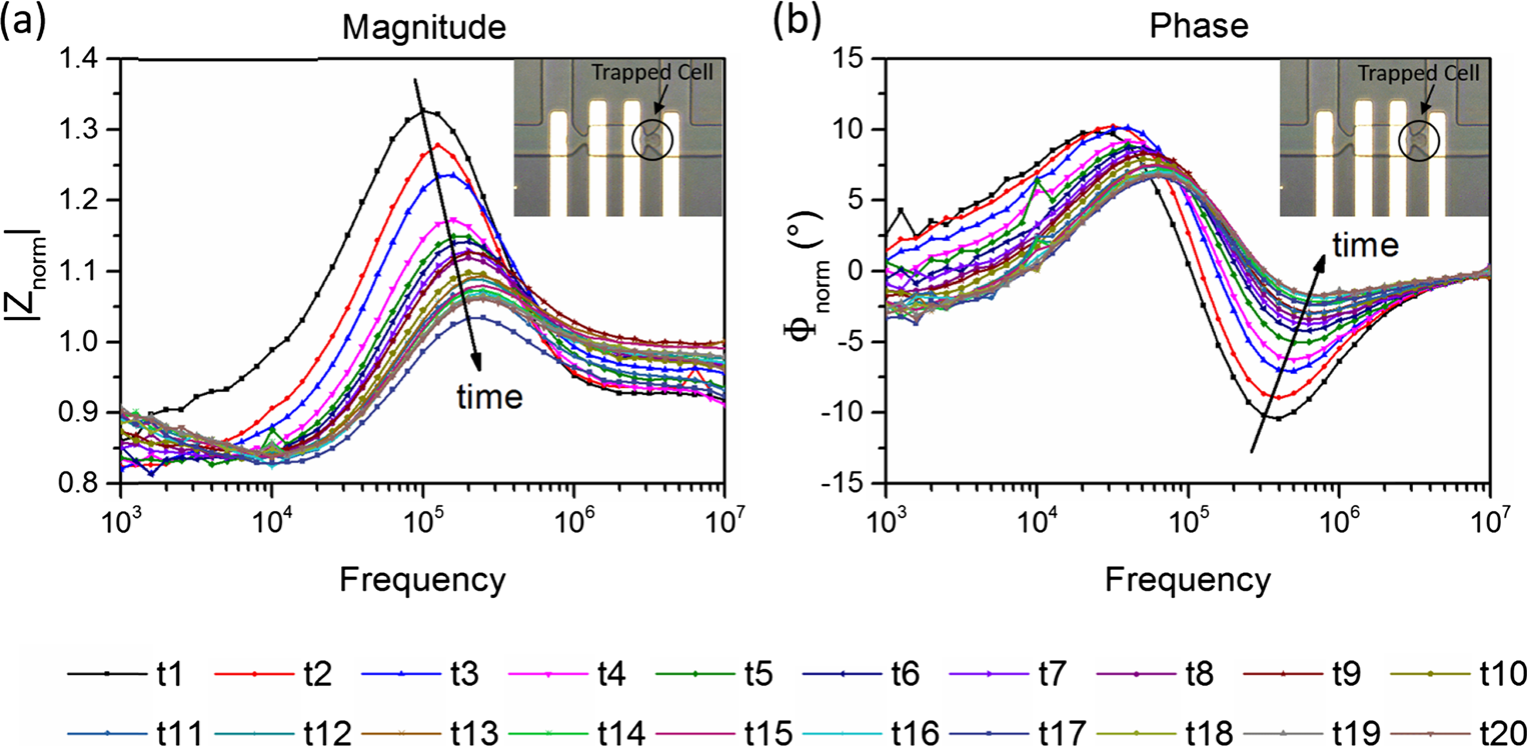
Real-time monitoring of single cell impedance change during membrane lysis. (a) Magnitude of the normalised impedance spectra. (b) Phase of the normalised impedance spectra. The embedded picture shows the particular cell that is being monitored. At time “t1”, impedance measurement was done for the normal cell, serving as a starting point. After that, Tween 20 was added into the device and triggered the membrane lysis process, and the impedance measurement was conducted every five minutes until “t20”. In total, twenty measurements were done in a time span of 95 min

The real-time response of a single cell during membrane lysis is shown in Fig. 4a (magnitude) and Fig. 4b (phase). The embedded microscope photo shows the particular cell that is being measured. At the time point “t1”, the impedance measurement of the cell at its normal state (i.e. before lysis) was performed, serving as a starting point and a reference. After that, the buffer solution with 0.5 % Tween 20 (Sigma-Aldrich) was added into the device, triggering the membrane lysis process. The impedance measurement was performed every five minutes until “t20”. In total, twenty measurements were done for the same cell in a time span of 95 min.

The most sudden change in cell impedance is observed in the first fifteen minutes after the cell is perfused with the lysis detergent, showing a 12 % decrease in the peak amplitude of the magnitude spectrum. A significant shift in the phase spectrum is also observed. After 15 mins, a decrease in the impedance magnitude can still be seen as time goes by, however, the decrease rate is slowing down. This trend of the impedance change during lysis is also observed for other cells tested. The decrease in impedance magnitude is caused by the pores formed in the cell membrane during lysis, thereby increasing the equivalent conductance of the membrane. As a result, the equivalent impedance of the whole cell decreases.

**Fig. 5:**
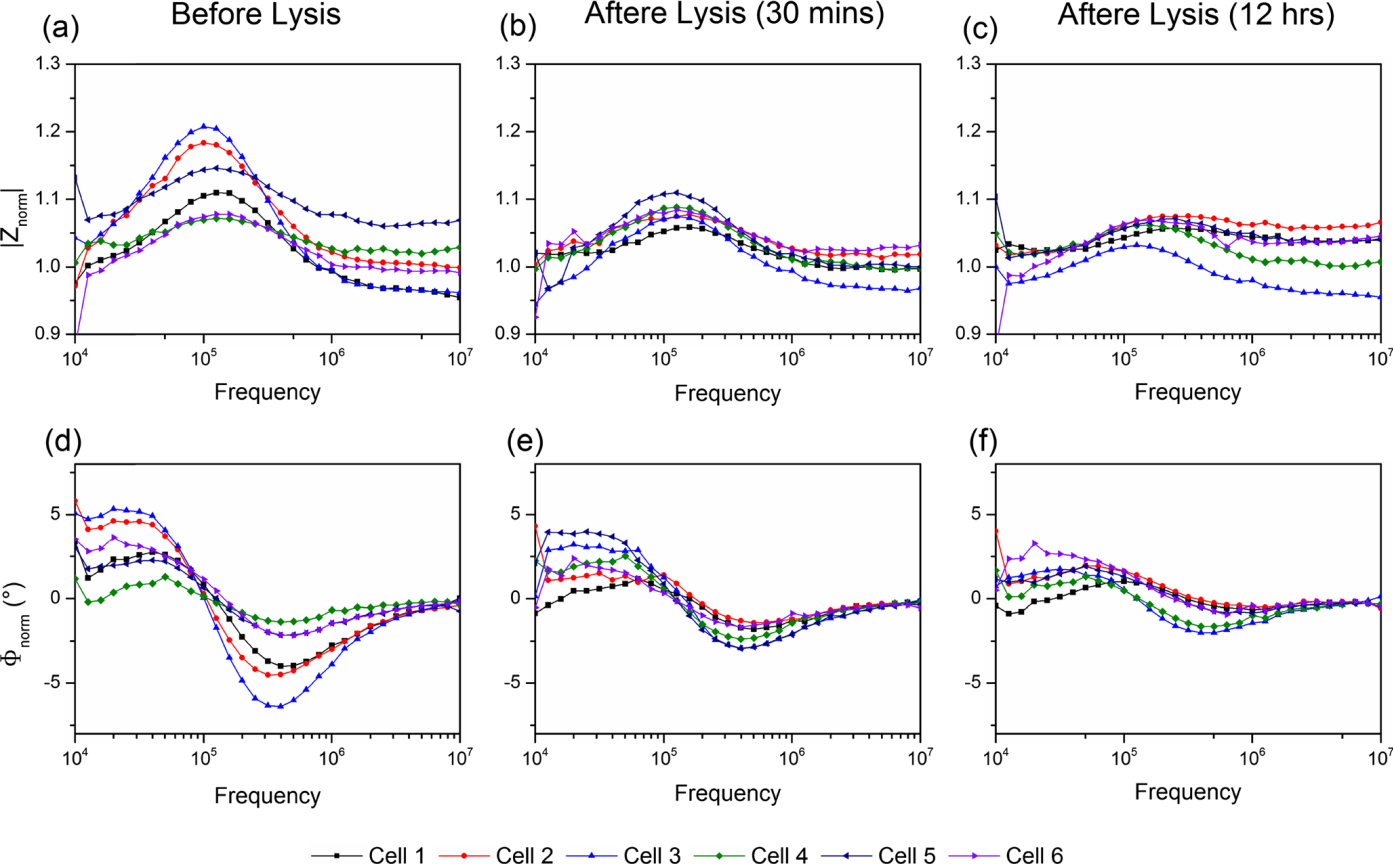
Examples showing the long-term monitoring of single cell impedance. Figures (a, b, c) are the magnitude, and figures (d, e, f) are the phase of the normalised impedance spectra. Six individual cells were characterised and monitored before lysis (a and d), 30 mins after lysis (b and e), and 12 h after lysis (c and f)

Figure 5 illustrates the long-term and simultaneous monitoring of multiple cells in different trap locations within the same device. The impedance spectra of six individual cells are shown here as examples. Figure 5 (a, b and c) show the magnitude, and (d, e, f) show the phase of the normalised impedance spectra. Cells were characterised and monitored before lysis (a and d), 30 mins after lysis (b and e), and 12 h after lysis (c and f). Even though single cells exhibit variations in impedance (also due to the fact that cells are not synchronised, i.e., cells are at different stages in a cell cycle and hence may have different properties), the changes in their impedance spectra follows the same trend during lysis. Both magnitude and phase (absolute value) tend to decrease. The impedance variations exhibited by different cells (i.e. heterogeneity) are largest when cells are normal (before lysis). After the membrane lysis, the variations become smaller, i.e., cells tend to a similar response. This indicates that the cell membrane plays an important role in determining the impedance of the cells and the unique dielectric signature of cells in the measurement range.

**Fig. 6:**
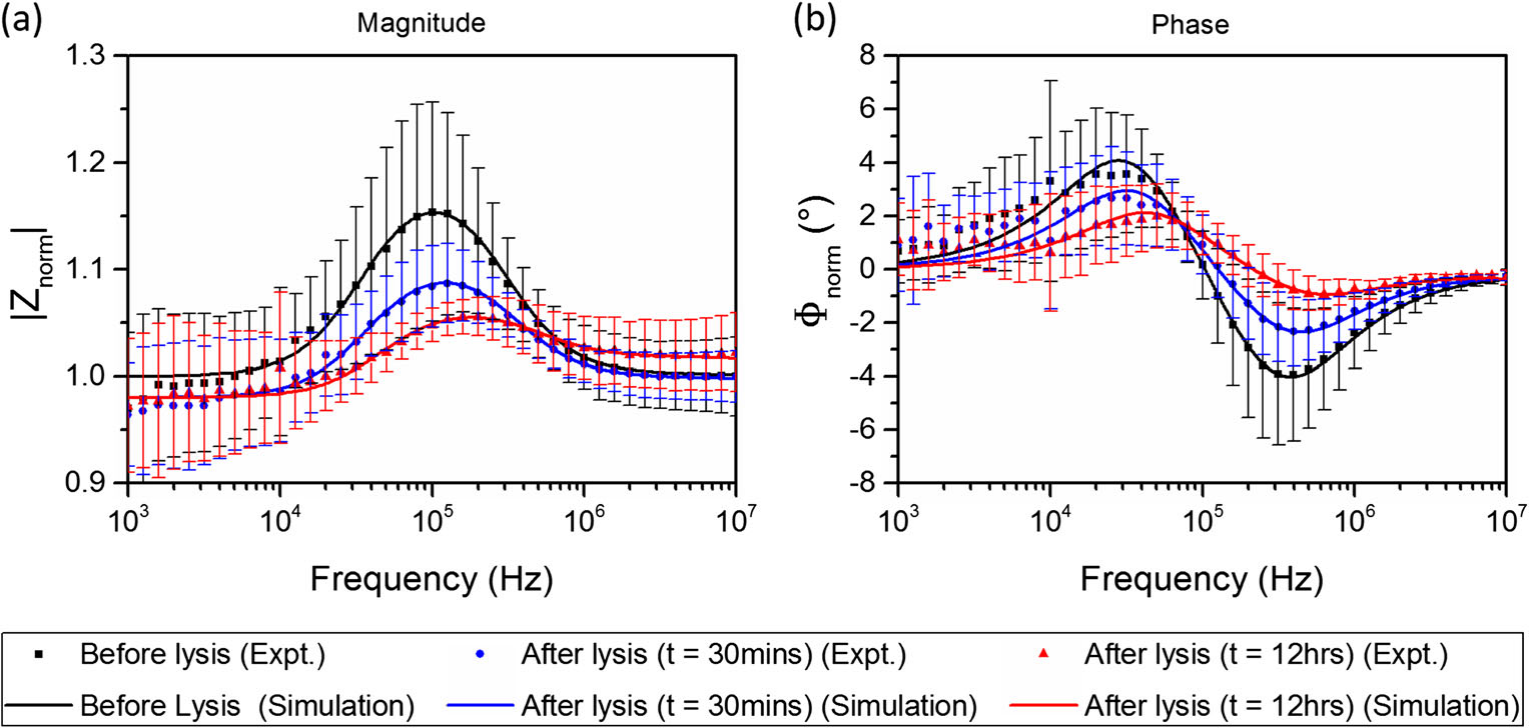
Averaged impedance magnitude (a) and phase (b) spectra of cells before and after lysis. The experimental data point at each frequency shows the average value for ten cells. Error bars show the standard deviation. The experimental data were fitted with the double-shell cell model

The averaged impedance spectra of cells before and after lysis are summarised in Fig. 6a (magnitude) and Fig. 6b (phase). The experimental data point at each frequency presents the average value of ten cells and error bar shows the standard deviation. The fitting procedure, based on double-shell model, was performed by assuming that the properties of the nucleus are unchanged during lysis. The assumption is based on the fact that Tween 20 is a relatively mild detergent, which can be used to lyse cell membrane, but is less efficient for permeabilising the nuclear membrane (Wu et al. 2006). Thus, the change in the nuclear membrane is assumed to be negligible compared with the change in cell membrane. The fixed parameters used in the fittings are: *R* = 6.2 μm; *d* = 6 nm; *d*_*ne*_= 40 nm; *σ*_*ne*_= 9.8 mS/m; *ε*_*ne*_= 5*ε*_0_; *ε*_*np*_= 60*ε*_0_; *ε*_*cp*_= 60*ε*_0_; N/C ratio = 0.78, where *R* is the outer radius of the cell, *d* and *d*_*ne*_ are the thicknesses of the cell membrane and nuclear envelope respectively, *σ*_*ne*_ and *ε*_*ne*_ are the conductivity and permittivity of the nuclear envelope, *ε*_*np*_ and *ε*_*cp*_ are the permittivities of the nucleoplasm and cytoplasm respectively, and N/C ratio is the nucleus-to-cytoplasm radius ratio.

The cell volume is kept constant during the fitting procedure to further simply the analysis. The cellular size of mouse embryonic stem cells was estimated by optical measurements under a microscope and the nuclear size was demonstrated previously by high-resolution fluorescence microscopy (Pagliara et al. 2014). Other parameters are extracted from the model. The extracted parameters are summarized in Table 1. The extracted parameters are generally comparable with those values mentioned in previous literature (Asami et al. 1989; Ermolina et al. 2000; Holmes et al. 2009; Mansor and Ahmad 2015; Polevaya et al. 1999). The difference between the values in this work and those stated in other works potentially indicates the unique electrical properties of the particular cell line (mESCs) being measured. For instance, the specific cell membrane capacitance measured here (0.025 F/m^2^) is higher than the values mentioned in some literature (0.006 F/m^2^), which may be explainable by the differences in the morphology of cell membranes, such as the extent of folding or surface features of the membrane (Muratore et al. 2012). In addition, the cell preparation procedures, such as cell fixation in formaldehyde, may also lead to a difference in the parameter values compared to those in literature.

**Table 1:** Electrical parameters of cells before membrane lysis (*t* = 0) and after membrane lysis (*t* = 30 mins; *t* = 12 h)

Lysis time	Membrane conductivity (μS/m)	Membrane specific capacitance (F/m^2^)	Cytoplasm conductivity (S/m)
*t* = 0	8	0.025 ± 0.006	0.48 ± 0.07
*t* = 30 mins	150	0.032 ± 0.007	0.38 ± 0.10
*t* = 12 h	500	0.041 ± 0.004	0.23 ± 0.07

As shown in Table 1, a significant increase in the equivalent conductivity of cell membrane is observed (8 μS/m before lysis; 150 μS/m after 30-min lysis; 500 μS/m after lysis for 12-h lysis) due to the pore formation in the membrane during lysis. The specific capacitance of the membrane tends to increase as well, which may be caused by an increase in the membrane permittivity, or a decrease in membrane thickness, or both. Another possible explanation for this is that the lysis detergent has altered cell membrane morphology or the membrane chemistry during lysis. The change in membrane morphology and chemistry can lead to a change in membrane capacitance (Muratore et al. 2012). On the other hand, the conductivity of the cytoplasm is observed to decrease (0.48 S/m before lysis; 0.38 S/m after lysis for 30 min; 0.23 S/m after lysis for 12 h), probably due to the fact that excess water enters the cytoplasm through the permeabilized cell membrane during lysis. However, it is still unknown to what extent the physicochemical properties of the cell membrane change as a result of the lysis process, as well as the particular cell preparation procedure (e.g cell fixation in formaldehyde). The research on investigating the changes in physicochemical properties of the cell membrane during lysis and the biological mechanism behind these changes is still ongoing.

It should be noted that even though the cell volume is kept unchanged in the fitting procedure, this may not be the true in in practice where an increase in cell volume has been observed during lysis (Malleo et al. 2010). The swelling of cells is caused by the excess water that moves into the cell through the disrupted membrane. It is expected that an increase in the cell volume will cause an increase in the impedance magnitude spectrum (based on simulations). However, the experimental results show an opposite trend – a decrease in cell impedance is observed. As mentioned above, the poration of the membrane can cause a decrease in cell impedance. This implies that the impedance decrease caused by the membrane poration must outweigh the impedance increase caused by cell volume increase. This also means that the extracted conductivities of the membrane after lysis in Table 1 may be underestimated, given that the cell volume is assumed unchanged during the fitting procedure. If there is an increase in the cell volume during lysis, the conductivity of the membrane must increase even more (than stated in Table 1) during the process to counteract the effects caused by the cell volume increase, so that the resulting overall impedance decreases, as verified by experimental results. If the change of cell size during the lysis process can be calibrated by combining other techniques such as optical methods, the uncertainty occurred during the electrical parameter fitting process would be decreased and the cell parameters would be extracted more accurately.

Nevertheless, the impedance-based single cell study quantitatively indicates how cell properties change during the lysis process. Multiple cells can be trapped and monitored simultaneously in the same device. Compared with traditional petri-dish based cell assays, the microfluidic device enables electrical impedance studies at the single cell level and monitoring their impedance spectra over a given period of time. Our preliminary studies reveal that cells can be trapped stably in the device over a significant time course. This would be beneficial to long-term culturing of single cells and analysis of cell dynamics over longer periods of time. In this work, we have demonstrated the usefulness of the device by monitoring single cell lysis. However, this device is not just limited to the measurement this particular process – it can also be used for real-time monitoring of other dynamic processes such as changes occurring during the cell cycle or during differentiation. Moreover, this device is also compatible with other detection methods such as fluorescence imaging techniques, opening up the possibility of integrating electrical and optical detection methods in a multiplexed format. Future work will incorporate the microfluidic device with external environmental control to investigate the dynamic changes in electrical properties of live cells.

## Conclusions

A PDMS-glass-based microfluidic device has been developed for single cell trapping and real-time impedance-based sensing. This platform enables the measurement of the actual frequency response of individual cells rather than the average among the cell population. Dynamic monitoring of mouse embryonic stem cells during cell lysis process has been demonstrated and quantitative analysis on the electrical properties of cells during this process is presented. Changes in cell membrane conductivity, specific capacitance and cytoplasm conductivity have been observed. The proposed device can be used to monitor and track time-dependent changes in electrical properties of individual cells, the analysis of which can shed light on the underlying mechanisms at a cellular level. This microfluidic platform also provides the possibility of performing analyses on large number of cells simultaneously to obtain data on statistical variability of the response. While a model system is presented here to validate device functionality, future work is expected to address real-time study of stem cell differentiation using the electrical impedance-based method including the quantitative analysis of changes in electrical properties during the cell cycle, as well as the investigation of how these changes can be correlated with specific biological mechanisms.
